# Initial management of patients with acquired aplastic anemia in the United States: results from a large national claims database

**DOI:** 10.1007/s00277-025-06307-z

**Published:** 2025-03-19

**Authors:** Jessica M. Stempel, Rong Wang, Alfred I. Lee, Amer M. Zeidan, Xiaomei Ma, Nikolai A. Podoltsev

**Affiliations:** 1 Section of Medical Oncology and Hematology, Department of Internal Medicine, Yale Comprehensive Cancer Center, Yale University School of Medicine, 333 Cedar Street, WWW208, New Haven, CT 06510 USA; 2 Cancer Outcomes, Public Policy, and Effectiveness Research (COPPER) Center, Yale University, New Haven, CT USA; 3 Department of Chronic Disease Epidemiology, Yale University School of Public Health, New Haven, CT USA

**Keywords:** Aplastic anemia, Paroxysmal nocturnal hemoglobinuria, Anti-thymocyte globulin, Eltrombopag, Real-world, Outcomes

## Abstract

Acquired aplastic anemia (AA) is an immune-mediated disorder leading to bone marrow failure characterized by pancytopenia, with infectious and bleeding complications. The disease course may be complicated by paroxysmal nocturnal hemoglobinuria (PNH), necessitating screening with flow cytometry (FC) at the time of AA diagnosis. Management strategies vary based on disease severity. Severe AA patients are usually heavily transfusion-dependent (HT-AA) and typically treated with antithymocyte globulin, calcineurin inhibitor (CNI) and eltrombopag (EPAG) as triple therapy, while allogeneic hematopoietic stem cell transplant (HSCT) is often reserved for younger patients with matched sibling donors. Moderate AA patients are less transfusion-dependent (LT-AA) and may be observed or treated with CNI or EPAG. We conducted a retrospective cohort study using Blue Cross Blue Shield Axis database, examining adult patients diagnosed with AA between 07/01/2016 and 06/30/2022. We evaluated their management within the first 6 months following the diagnosis. Of 793 identified individuals (542 LT-AA, 251 HT-AA), with a median age of 49 years, only 42.6% received AA-directed therapy. Triple therapy and HSCT were infrequently used for patients with HT-AA (4.4% and 18.7%, respectively), while the most common treatment was the combination of a CNI and EPAG (LT-AA 37.8%, HT-AA 51.7%). The median time from diagnosis to treatment was 22 days, with older patients (age ≥ 40 years) experiencing treatment initiation delays (*p* = 0.03). FC testing was underutilized with only 55.5% of patients undergoing evaluation. These findings highlight the need for better access to diagnostic evaluation and appropriate AA-directed therapy for patients with AA in real-world settings.

**Supplementary Information:**

The online version contains supplementary material available at 10.1007/s00277-025-06307-z.

## Introduction

Acquired aplastic anemia (AA) is an exceedingly rare immune-mediated disease with a reported incidence ranging between 2–3 cases per million per year [1]. It is characterized by cytotoxic and cytokine-mediated destruction of the hematopoietic and progenitor cells, and ultimately bone marrow failure, pancytopenia, and an increased risk of infection, bleeding and death [2, 3]. Notably, 40–60% of patients with AA harbor paroxysmal nocturnal hemoglobinuria (PNH) clones detectable by blood flow cytometry, and in some cases, can progress to clinically significant PNH necessitating anti-complement therapy [4]. Allogeneic hematopoietic cell transplantation (HSCT) is the only curative therapy for AA, primarily offered in first-line to patients with severe disease who are under 40 years of age and have a matched sibling donor [5].

Horse-derived anti-thymocyte globulin (ATG) became the backbone of AA-directed therapy nearly 50 years ago, following observations of improved hematopoiesis and overall survival (OS) [6, 7]. Early attempts to improve outcomes with ATG combinations were unsuccessful until the addition of cyclosporine (CsA), a calcineurin inhibitor (CNI), which demonstrated superior OS in patients with AA managed with ATG [8]. The subsequent addition of eltrombopag (EPAG), a thrombopoietin receptor agonist which stimulates hematopoiesis of all cell lineages [9], further transformed the treatment landscape. EPAG added to immunosuppressive therapy (IST, ATG and CsA) improved the complete response (CR) rate at 3 months, the 6-month overall response rate (ORR), and shortened the median time to response when compared to IST alone in patients with severe AA [10]. These results supported the Food and Drug Administration’s approval (2018) of EPAG in IST as first-line treatment for individuals with severe AA and has become the standard of care treatment for patients who are not candidates for HSCT [11–15].

Due to rarity of AA, previous studies are limited to a small number of clinical trials and observational studies from select tertiary care centers. This limited scope does not allow the comprehensive assessment of real-world patterns of care, including the selection of the first-line therapies. To address this gap, we examined the treatment patterns and outcomes among adult patients with newly diagnosed AA using the Blue Cross Blue Shield (BCBS) Axis administrative database, which captures commercial healthcare claims of approximately one third of the United States (US) population and provides a robust platform for evaluating real-world management practices [16].

## Methods

In this observational claims-based study, we focused on the initial work-up and management of adult patients with AA within the first 6 months after diagnosis using the BCBS Axis database [16]. We included patients with at least one inpatient diagnosis of AA or at least two outpatient diagnoses of AA more than 30 days apart. AA diagnosis was based on the International Classification of Diseases 10th edition (ICD-10) D61.3, D61.89, D61.9. Additionally, we required patients to meet the following criteria: 1) ≥ 18 years of age, 2) newly diagnosed AA during 07/01/2016—06/30/2022, and 3) covered by BCBS continuously from 6 months before to 6 months after diagnosis of AA, including prescription drug coverage. Patients were excluded if they had unknown baseline characteristics, or had a diagnosis of acute myeloid leukemia or myelodysplastic syndrome/neoplasm within 6 months before or after AA diagnosis. We categorized patients as having higher transfusion requirement AA (HT-AA) and lower transfusion requirement AA (LT-AA). We defined HT-AA as patients who received at least 4 units of blood product transfusions (red blood cells and/or platelets) over an 8-week period prior to AA diagnosis, underwent HSCT and/or received ATG during the observation period of 6 months after the diagnosis of AA. All other patients, including less frequently transfused and transfusion-independent during 8-week period prior to AA diagnosis, were assigned to the LT-AA group.

We evaluated hospitalization metrics including the rates of hospitalization, reasons for hospital admission and emergency department (ED) visits, and length of hospital stay within the first 6 months of AA diagnosis. To assess outcomes of interest, we utilized ICD-10 diagnosis codes to identify infectious and bleeding complications (ICD-10 codes listed in Supplemental Materials, Table S1). We investigated how many patients were tested with peripheral blood flow cytometry necessary to diagnose PNH using respective codes, provided in Table S1. We identified AA-directed treatment and supportive care interventions by ICD-10 procedure codes, Healthcare Common Procedure Coding System codes and National Drug codes. AA-directed treatments and supportive-care medications are listed in TableS2.

Descriptive statistics were generated for all variables and outcomes of interest, including frequency distributions for categorical variables, and mean, standard deviation, and median (and percentiles) for continuous variables. We compared baseline and management characteristics of patients with LT-AA and HT-AA. We used χ^2^ test for categorical variables and Student t-test or the Mann–Whitney test for continuous variables, as appropriate. We used multivariable logistic regression analysis to assess which patient- and regional-level factors influence treatment decision for the initial therapy of AA. We used multivariable Cox proportional hazards model to evaluate factors impacting time-to-treatment initiation (TTT) of AA-directed treatments. All statistical tests were two-sided with a type I error of 0.05 and conducted using R Version 4.2.2 (The R Project for Statistical Computing). The Yale Human Investigation Committee determined that this study did not directly involve human subjects.

## Results

**Fig. 1:**
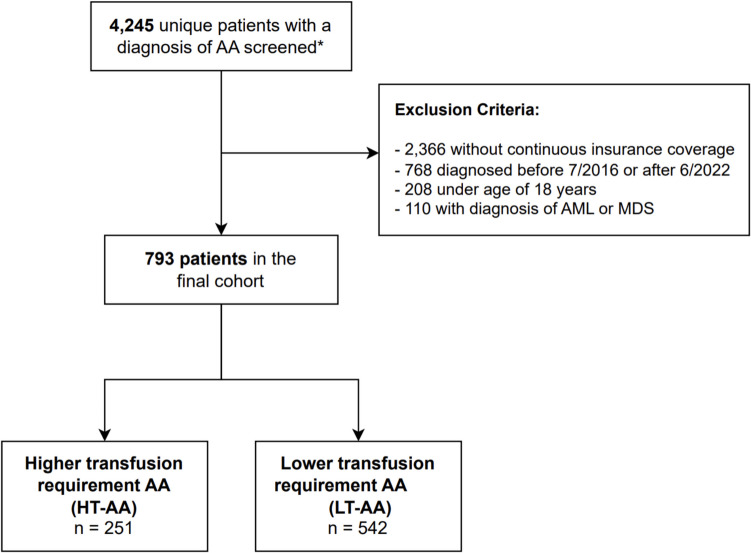
**Cohort Selection. **AA: aplastic anemia; AMD/MDS: acute myeloid leukemia/myelodysplastic syndromes. * Met criteria for one inpatient or 2 outpatient diagnostic codes for AA at least 30 days apart

We identified 4,245 individuals with a diagnosis of AA in the BCBS database. After implementing the eligibility criteria, a total of 793 patients were included in the final cohort (Fig. 1). The median age of diagnosis was 49 (interquartile range [IQR], 33–59) years and 52.3% of patients were female. 271 (34.2%) patients were under the age of 40 years. When examined by social deprivation index (SDI), 318 (40.1%) patients were in the 1st tertile (corresponding to the highest socioeconomic status), whereas 184 (23.1%) were in the lowest (3rd) tertile.

We identified 542 (69.2%) patients with LT-AA and 251 (30.8%) patients with HT-AA. There were no significant differences in the sociodemographic characteristics between both groups (Table 1). More patients with LT-AA had lower Elixhauser comorbidity scores (lower comorbidity burden) compared to patients with HT-AA (24.2% vs 15.1%, *p* = 0.01).

**Table 1:** Patient characteristics of 793 patients with aplastic anemia

Variables	LT-AA *n* = 542 (%)	HT-AA *n* = 251 (%)	*p*-value
Age at diagnosis, median (IQR)	50 (35–60)	48 (30–59)	0.07
Age < 40 (%)	173 (31.9)	98 (39.0)	0.14
Female	289 (53.3)	126 (50.2)	0.46
Social deprivation index			
1st tertile (low deprivation, high SES)	212 (39.1)	106 (42.2)	0.29
2nd tertile	201 (37.1)	79 (31.5)	
3rd tertile (high deprivation, low SES)	121 (22.3)	63 (25.2)	
Metropolitan area resident	439 (81)	208 (82.9)	0.59
US region			
Northeast	78 (14.4)	45 (17.9)	0.32
Midwest	114 (21)	54 (21.5)	
South	244 (45)	97 (38.6)	
West	106 (19.6)	55 (21.9)	
Elixhauser score			
0	131 (24.2)	38 (15.1)	0.01
1–2	171 (31.5)	95 (37.8)	
≥ 3	240 (44.3)	118 (47)	

*HT-AA * higher transfusion requirement AA, *LT-AA *lower transfusion requirement AA, *IQR *interquartile range, *SES* socioeconomic status, *US* United States

### AA-directed treatment within the first 6 months of AA diagnosis

A total of 338 (42.6%) patients in our cohort received AA-directed therapy within 6 months from the initial diagnosis (Table 2). Only HT-AA patients received HSCT and ATG, according to our predefined patient categories. Overall, AA-directed treatment was used for 203 patients (80.9%) with HT-AA and 138 patients with LT-AA (24.9%). CNI were utilized in 295 patients (CsA, *n* = 234; tacrolimus, *n* = 77; with 16 having received both at different times over a 6-month period). CNI was similarly prescribed among patients with HT-AA (89.9%) and LT-AA (84.4%) (*p* = 0.20), and EPAG was more frequently prescribed to patients with HT-AA (68.5% vs 49.9%, *p* < 0.01).

**Table 2:** Aplastic anemia-directed treatment and supportive care

Variables	LT-AA *n* = 542 (%)	HT-AA *n* = 251 (%)	*p*-value
AA-directed treatment ^a^	**135 (24.9)**	**203 (80.9)**	
HSCT	0	38 (18.7)	-
ATG	0	15 (7.4)	-
CNI	114 (84.4)	181 (89.2)	0.20
EPAG	66 (48.9)	139 (68.5)	< 0.01
Treatment combinations ^b^			
CNI + EPAG	50 (37.0)	104 (51.2)	0.01
CNI + EPAG + ATG	0	9 (4.4)	-
Supportive care	**287 (53.0)**	**251 (100)**	
RBC transfusions ^c^	107 (37.3)	235 (93.6)	< 0.01
Platelet transfusions ^c^	36 (12.5)	192 (76.5)	< 0.01
Antimicrobials ^c^	213 (74.2)	203 (80.9)	0.07
PNH-directed therapy	15 (2.8)	10 (4.0)	0.36

*AA* aplastic anemia, *HT-AA*: higher transfusion requirement AA, *LT-AA* lower transfusion requirement AA, *HSCT* allogeneic hematopoietic cell transplant, *ATG* antithymocyte globulin, *CNI* calcineurin inhibitor (cyclosporine A or tacrolimus), *EPAG* eltrombopag, *RBC* red blood cells, *PNH* paroxysmal nocturnal hemoglobinuria

^a^ Treatment categories are not mutually exclusive

^b^ Concurrent treatment exclusively, CNI and EPAG started within 30 days of each other^c^ Percentages based on those who received supportive care

The most frequently prescribed treatment combination was CNI and EPAG in 154 patients (LT-AA, *n* = 50 [37.0%]; HT-AA, *n* = 104 [51.2%], *p* < 0.01) (Table 2). Among individuals with HT-AA who received AA-directed treatment, triple therapy was reported in only 9 patients (4.4%).

HSCT was reported in 38 patients (18.7%) with HT-AA of which 26 were < 40 years. The median time to transplant was 85 (IQR 43.5–116) days. The majority of these patients received CNI alone around the time of diagnosis (24 of 38, 63.2%), and 9 of 38 patients (23.7%) received EPAG plus CNI prior to proceeding to HSCT.

We examined factors among the entire cohort which could impact the initial treatment decision. When adjusting for age, comorbidities, SDI, and region of residence, patients with HT-AA were more likely to receive AA-directed treatment within 6 months of AA diagnosis compared to LT-AA (adjusted odds ratio [OR] = 13.32, 95% confidence interval [CI]: 9.17–19.70, *p* < 0.01) (Table 3). Comorbidity burden was not and independent predictor of receiving AA-directed treatment (Elixhauser score 1–2 vs 0: OR = 1.20, 95% CI 0.75–1.92, *p *= 0.45; Elixhauser score ≥ 3 vs 0: OR = 0.95, 95% CI: 0.60–1.53, *p* = 0.84).

**Table 3:** Factors associated with receipt of aplastic anemia-directed treatment

	Univariate			Adjusted		
	OR	95% CI	p	OR	95% CI	p
Higher-transfusion requirement AA	12.75	8.88–18.62	< .01	13.32	9.17–19.70	< .01
Age at diagnosis						
18–39	1.00			1.00		
40–64	0.79	0.58–1.06	0.12	0.92	0.63–1.35	0.68
65 +	0.46	0.23–0.88	0.02	0.43	0.19–0.96	0.04
Male	1.13	0.85–1.50	0.40	1.10	0.78–1.54	0.59
Elixhauser score						
0	1.00			1.00		
1–2	1.54	1.04–2.29	0.03	1.20	0.75–1.92	0.45
3 +	1.19	0.82–1.73	0.37	0.95	0.60–1.53	0.84
Non-metropolitan area residence	0.89	0.62–1.29	0.55	0.90	0.57–1.40	0.64
Social deprivation index						
1st tertile (highest SES)	1.00			1.00		
2nd tertile	0.82	0.59–1.13	0.23	0.96	0.65–1.44	0.85
3rd tertile (lowest SES)	0.78	0.54–1.13	0.20	0.74	0.46–1.16	0.19
Unknown	1.43	0.42–5.06	0.56	2.28	0.53–9.61	0.26
US region						
Northeast	1.00			1.00		
Midwest	1.32	0.83–2.11	0.24	1.63	0.93–2.88	0.09
South	0.83	0.55–1.27	0.40	1.05	0.63–1.76	0.87
West	0.99	0.62–1.59	0.97	1.08	0.61–1.93	0.78

*AA* aplastic anemia, *CI* confidence interval, *OR *odds ratio, *SES* socioeconomic status, *US* United States

Based on multivariable analysis, individuals ≥ 65 years were less likely to receive treatment for AA compared to younger patients (< 40 years) (OR = 0.43, 95% CI: 0.19–0.196, *p* = 0.04). As HT-AA patients included HSCT and/or ATG recipients, we conducted further analysis excluding individuals who received HSCT and ATG (treatments defining patients as belonging to HT-AA group) (data not shown). As a result, the impact of older age (≥ 65 years) on the initiation of AA-directed treatment lost its significance (OR = 0.48, 95% CI: 0.21–1.07, *p* = 0.08).

**Fig. 2:**
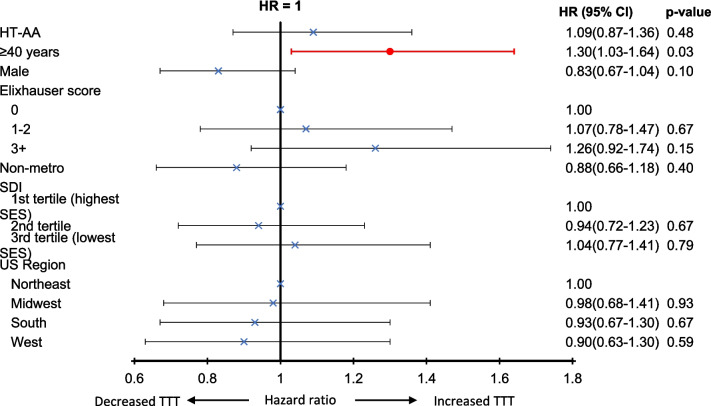
Factors impacting time-to-treatment initiation of aplastic anemia-directed therapy among 338 treated patients with aplastic anemia*. *Among patients who received treatment for AA. HT-AA: higher transfusion requirement AA; SDI: social deprivation index; SES: socioeconomic status; US: United States; TTT: time to treatment initiation; HR: hazards ratio

The median TTT after AA diagnosis was the same in the LT-AA and HT-AA groups, 22 days (IQR: 10–49 days). Older patients (≥ 40 years) had an increased TTT when compared to younger patients (hazard ratio = 1.30, 95% CI: 1.03–1.64), *p* = 0.03). Comorbidity index, demographic, socioeconomic or regional factors did not impact TTT **(**Fig. 2**)**.

### PNH testing and treatment

**Fig. 3:**
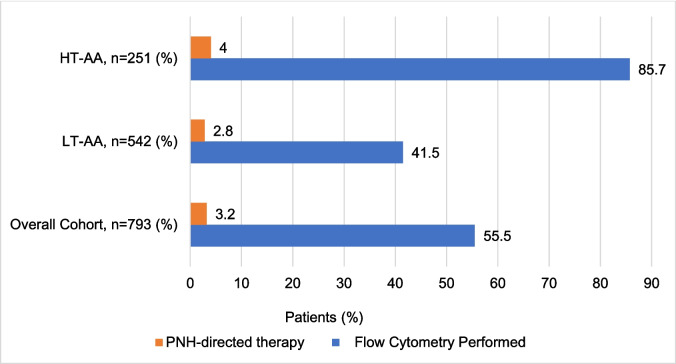
**Paroxysmal nocturnal hemoglobinuria testing and treatment**. HT-AA: higher transfusion requirement AA; LT-AA: lower transfusion requirement AA; PNH: paroxysmal nocturnal hemoglobinuria

Across the entire cohort of individuals with AA, only 440 (55.5%) had any FC testing performed around the time of AA diagnosis, of which 215 (85.7%) had HT-AA and 225 (41.5%) had LT-AA (*p* < 0.01). 25 (3.2%) patients with AA were prescribed anticomplement therapy within 6 months of AA diagnosis (Fig. 3).

### Supportive care

The majority of patients in our cohort received supportive care (538, 67.8%), including all patients with HT-AA, per our pre-established definition. Antimicrobial therapy was prescribed to 416 (77.2%) patients. Among patients who received supportive care interventions, red blood cell transfusions were given to 107 (37.3%) and 235 (93.6%) of patients with LT-AA and HT-AA, respectively. Platelet transfusions were reported in 36 (12.5%) patients with LT-AA and 192 (76.5%) patients with HT-AA. The median number of transfusion days after AA diagnosis for patients with HT-AA was 6 (IQR: 4–10) days for RBCs and 7 (IQR: 4–12.2) days for platelets (Table 2). 24 (8.4%) patients with LT-AA and 45 (17.9%) with HT-AA were given granulocyte stimulating agents. Erythrocyte stimulating agents were utilized in 20 (7.0%) patients with LT-AA and 8 (3.2%) with HT-AA.

### Hospital utilization and complications of AA

Within 6 months after AA diagnosis, at least one hospital admission was reported in 397 patients (50.1%). Patients with LT-AA had fewer inpatient admissions (195, 36.0%) and emergency department visits (70, 12.9%) compared to patients with HT-AA (202, 80.5% and 95, 37.8%, respectively; *p* < 0.01). The inpatient median length of stay (days) was significantly greater for patients with HT-AA (16, IQR: 7–39) than for patients with LT-AA (6, IQR: 3–14) (*p* < 0.01). Table 4 provides comprehensive details on hospitalization characteristics and associated complications.

**Table 4:** Hospitalizations, bleeding and infectious complications

Variables	LT-AA *n* = 542 (%)	HT-AA *n* = 251 (%)	*p*-value
Infections	106 (19.6)	111 (44.2)	< 0.01
Bleeding	135 (24.9)	124 (49.4)	< 0.01
Genitourinary^a^	44 (32.6)	50 (40.3)	0.20
Gastrointestinal^a^	54 (40.0)	30 (24.2)	0.01
Respiratory^a^	20 (14.8)	45 (36.3)	0.01
Hospitalizations	195 (36.0)	202 (80.5)	< 0.01
Hospitalizations due to infection ^b^	28 (14.4)	47 (23.3)	0.03
Hospitalizations due to bleeding^b^	28 (14.4)	40 (19.8)	0.19
Hospitalizations due to AA^b^	114 (58.5)	165 (81.7)	< 0.01

*HT-AA* higher transfusion requirement AA, *LT-AA* lower transfusion requirement AA

^a^ Percentages based on those with bleeding complications

^b^ Percentages based on those with inpatient visits

## Discussion

To our knowledge, this study represents one of the largest real-world analyses of adult patients with AA in the US. We examined treatment patterns and management characteristics of newly diagnosed patients with this disease since the approval of EPAG as part of the first-line triple therapy. We report that the combination of CNI and EPAG was the most frequently used treatment regimen for patients with HT-AA, significantly exceeding the utilization of the recommended first-line triple therapy (51.7% vs 4.9%). Notably, an ATG-free approach with CsA and EPAG was recently evaluated in a single-arm phase 2 study of individuals with severe AA who had limited access to ATG or were at increased risk for ATG-related toxicity [17]. This regimen was well tolerated, with 25 patients (46%) achieving an ORR at 6 months, including 23 partial responses and but only 2 patients with CR. While these outcomes are less favorable compared to those observed with triple therapy, ATG-free strategies are appealing to both patients and physicians, mainly due to the convenience of an oral-only therapy and avoidance of the risks associated with the administration of ATG including anaphylaxis, serum sickness, and need for inpatient admission [18]. Despite these considerations, as ATG is available in the US, efforts should be made to initiate the optimal evidence-based approach for eligible patients. Therefore, most of the patients who are starting the treatment with EPAG and CNI should be considered for triple therapy.

HSCT for severe AA results in high cure rates and low risk of graft-versus-host disease, particularly in younger patients (< 40 years). HSCT also reduces the risk for disease relapse, and the development of PNH and myeloid malignancies [5, 19]. Current guidelines emphasize the early transplant referral to minimize disease-related complications[14, 15]. Traditionally, transplant eligibility has been dichotomized by age (< / ≥ 40 years); however, advancements in transplant techniques and therapies are challenging this threshold, making age a less stringent determinant and enabling a more individualized assessment of transplant eligibility [15, 20, 21]. This study revealed that the overall utilization of HSCT was low (18.7%) with only 9.6% of individuals younger than 40 years undergoing HSCT in the initial 6 months of diagnosis. While the small number of patients undergoing HSCT suggests potential barriers to timely transplantation—such as lack of access to specialized care, donor unavailability, and patient-level factors—our dataset does not provide sufficient granularity to evaluate the reasons for forgoing HSCT. These findings highlight the need for further research to understand and address potential barriers for the use of HSCT for the treatment of AA.

Up to 60% of patients with acquired AA can harbor a PNH clone which may become clinically significant during follow up [4]. This rare acquired disorder could lead to complement-mediated hemolysis, increased risk for thrombosis, and organ disfunction. Screening and monitoring of PNH by peripheral blood flow cytometry is a crucial component of the management of AA and is recommended at the time of AA diagnosis for all patients, regardless of disease severity. [14, 15] In our analysis, only 55.5% of patients had at least one flow cytometry test within 6 months of the AA diagnosis. Testing was more frequently performed in the HT-AA subgroup (85.7%) in contrast to the LT-AA subgroup in which only 41.5% of patients had a flow cytometry test. Our results highlight the potential misperception of PNH risk in less cytopenic AA patients leading to lower testing frequency in this subgroup, and overall, a significant underutilization of PNH screening in the adult AA population.

In this national database, patients ≥ 40 years of age were more likely to experience delays in treatment initiation. Comorbidity burden on the other hand, did not have impact on the likelihood to receive AA-directed therapy or TTT. For both LT-AA and HT-AA patient groups, the median time to initiation of AA-directed therapy was 22 days. Non-IST-based approaches, including supportive care alone, were primarily utilized for older patients with HT-AA as well as those with LT-AA. The BCBS database covers a younger patient population therefore limiting the generalizability to patients ≥ 65 years. In our study, only 48 patients (6.1%) were in this older age group, further challenging the applicability of these results to an older population.

We present one of the largest outcomes cohorts of adult patients with AA in the US and the sample size of our study is a unique strength taking into consideration the very low incidence of this disease [22–25]. We identified individuals in the BCBS Axis database, which has not been previously utilized for investigating the initial management of patients with AA. Real-world cohorts of patients with AA often include pediatric populations and are limited to a single institution or handful of larger tertiary medical centers specialized in management of AA. This study provides valuable insights into the patters of care delivered to adults with AA in the real-world conventional care settings. The observed underutilization of treatment for patients with AA raises important considerations, as reflected in the low rates a triple-therapy and HSCT. Patient-related factors such as advanced age, increased number of comorbidities, or poor functional status, may limit eligibility for intensive treatments due to the higher risk of complications. Systemic barriers, including limited access to specialized healthcare, donor availability (for transplant-eligible patients), and financial constraints, also likely lead to treatment gaps. Lastly, variability in physician familiarity with AA treatment guidelines and different levels of comfort in recommending aggressive interventions may influence treatment patterns and decision-making. Further research is needed to address patient-, system-, and provider-level factors to improve access to and utilization of evidence-based therapies for AA.

This study has several important limitations. BCBS Axis claims data does not contain laboratory values, pathology, or molecular testing results. The lack of this granular clinical data prevents the independent confirmation of the diagnosis of AA and limits our ability to classify disease severity according to the well-established Camitta criteria, which rely on specific threshold for marrow cellularity and peripheral blood counts [26]. Given that patients with severe and very severe AA are more commonly transfusion-dependent than those with moderate AA, [27] we classified patients based on the number of transfusion received during 8 weeks prior to AA diagnosis (LT-AA and HT-AA) as a pragmatic alternative to stratify disease severity. These criteria, while practical and adapted to the available data, differ from the conventional criteria, highlighting the need for caution while interpreting disease severity in the context of this study. The dataset also limits the ability to confirm the indication for flow cytometry which may overestimate the frequency of PNH testing in our study. Data surrounding patient demographics is also limited and thus we are unable to identify differences in treatment based on race and ethnicity. Additionally, our observation period began one year before the FDA approval of triple therapy for AA and may have contributed to the lower number of patients utilizing this treatment approach. Our results have limited applicability to pediatric and older patient population.

Our study offers critical insights into the real-world initial management of patients with AA in the US, revealing a potential underutilization of PNH testing, triple therapy and HSCT, and the higher risk for treatment delays in older patients with HT-AA. Our results emphasize the need for better access to appropriate diagnostic evaluation and AA-directed treatment.

## Supplementary Information

Below is the link to the electronic supplementary material. 

ESM1 (DOCX 17.9 KB)

## Data Availability

This study used the Blue Cross Blue Shield Axis Database, which is a proprietary database acquired from a third party (Blue Cross Blue Shield Association). There are legal restrictions in the conditions of our data use agreement prohibiting us from sharing the data publicly. Investigators wishing to obtain the Blue Cross Blue Shield Axis Database would be able to access these data by contacting Blue Cross Blue Shield Association.
