# Supplementary material for: Initial management of patients with acquired aplastic anemia in the United States: results from a large national claims database
Source: Ann Hematol. Author manuscript; Available in PMC 2025 May 7. (PMC12052795; doi:10.1007/s00277-025-06307-z)
Supplement: Supplemental material [file NIHMS2078203-supplement-Supplemental_material.docx]

**Initial management of patients with acquired aplastic anemia in the United States: results from a large national claims database**

**Annals of Hematology**

Jessica M. Stempel^1,2^, Rong Wang^2,3^, Alfred Ian Lee^1^, Amer M. Zeidan^1,2^, Xiaomei Ma ^2,3*^, Nikolai A. Podoltsev^1,2*^

Affiliations:

^1^Section of Medical Oncology and Hematology, Department of Internal Medicine, Yale School of Medicine, and Yale Comprehensive Cancer Center, New Haven, CT

^2^Cancer Outcomes, Public Policy, and Effectiveness Research (COPPER) Center, Yale University, New Haven, CT

^3^Department of Chronic Disease Epidemiology, Yale University School of Public Health, New Haven, CT

* Co-senior authors

**Corresponding Author:**

**Jessica M Stempel MD**

[Jessica.stempel@yale.edu](mailto:Jessica.stempel@yale.edu)

**Supplementary Material**

**Table S1. List of ICD-10 codes used for the bleeding and infectious complications of interest.**

|  | **ICD-10** |
| --- | --- |
| **Infectious Complications** | A04.7, A15, A17, A19, A31, A39-A43, A46, A48.0, A48.8, A49, A83, A85-A87, A89, B00-B02, B10, B20, B25, B27.1, B27.8, B27.9, B33, B34, B37-B39, B44, B49, B58- B60, B64, B95, B96, B97.0, B97.1, B97.2, B97.4, B97.6, B97.8, B99 |
| **Bleeding Complications** | |
| **Eye/Ear** | H11.3, H21.0, H31.3, H35.6, H43.1, H45.0, H92.2 |
| **Cardiac** | I23.0, I31.2, J94.2 |
| **Joints** | M25.0 |
| **Genitourinary** | R31, N02, N42.1, N92.0, N92.1, N92.3, N92.4, N93.0, N93.8, N93.9, N95.0 |
| **Respiratory** | R04 |
| **Gastrointestinal** | I85.0, K25.0, K25.2, K25.4, K25.6, K26.0, K26.2, K26.4, K26.6, K27.0, K27.2, K27.4, K27.6, K28.0, K28.2, K28.4, K28.6, K29.0, K62.5, K66.1, K92.0, K92.1, K92.2 |
| **Central nervous** | I60, I61 |
| **Other** | D62, D68.3, D69.8, D69.9, R58, S06.4 -S06.6, S26.0, S27.1, T79.2 |

ICD: International Classification of Diseases 10^th^ edition

**Table S2. AA-directed treatment list.**

|  | **Treatment (agents) for patients with aplastic anemia** |
| --- | --- |
| *AA-directed* | Allogeneic hematopoietic stem cell transplant (HSCT) |
|  | Anti-Thymocyte Globulin (ATG):  Lymphocyte immune globulin, antithymocyte (equine),   Lymphocyte immune globulin, antithymocyte (rabbit) |
|  | Calcineurin inhibitors: Cyclosporine, Tacrolimus |
|  | Thrombopoietic agents: Eltrombopag, Romiplostim |
|  | Alemtuzumab |
|  | Cyclophosphamide |
|  | Androgens: Danazol, Fluoxymesterone, Methyltestosterone, Nandrolone,  Oxandrolone, Oxymetholone, Stanozolol |
| *Supportive care* | Erythropoiesis-stimulating agent: Epoetin, Darbepoetin |
|  | Granulocyte stimulating factors: Filgrastim, Pegfilgrastim, Sargramostim |
|  | Red blood cell and platelet transfusion |
|  | Anti-fungal, antiviral, and antibacterial agents |
